# Insights into pH regulatory mechanisms in mediating spermatozoa functions

**DOI:** 10.14202/vetworld.2018.852-858

**Published:** 2018-06-26

**Authors:** Abhishek Kumar Mishra, Akshay Kumar, Dilip Kumar Swain, Sarvajeet Yadav, Rajesh Nigam

**Affiliations:** 1 College of Biotechnology, U.P. Pandit Deendayal Upadhayaya Pashu Chikitsa Vigyan Vishwavidyalaya Evam Go Anusandhan Sansthan, Mathura - 281 001, Uttar Pradesh, India; 2 Department of Gynaecology and Obstetrics, College of Veterinary Sciences and Animal Husbandry, U.P. Pandit Deendayal Upadhayaya Pashu Chikitsa Vigyan Vishwavidyalaya Evam Go Anusandhan Sansthan, Mathura - 281 001, Uttar Pradesh, India; 3 Department of Physiology, College of Veterinary Sciences and Animal Husbandry, U.P. Pandit Deendayal Upadhayaya Pashu Chikitsa Vigyan Vishwavidyalaya Evam Go Anusandhan Sansthan, Mathura - 281 001, Uttar Pradesh, India

**Keywords:** acrosome reaction, capacitation, fertilization, pH, sperm motility, spermatozoa

## Abstract

Regulation of pH in spermatozoa is a complex and dynamic process as sperm cells encounter different pH gradients during their journey from testes to the site of fertilization in female genital tract. The precise regulations of pH in sperm cells regulate the sperm functions such as motility, hyperactivity, capacitation, and acrosome reaction. Electrophysiological, pharmacological, and molecular studies have revealed the presence of different ion channels and exchanger systems which regulate intracellular pH in sperm cells as well as regulate sperm functions. Recent studies also have shown the potential involvement of pH in the regulation of fertility competence of sperm cells, and alterations in pH have shown to impede sperm functions. This mini-review discusses the probable mechanisms involved in pH regulation in sperm cells and how pH is involved in regulation of various sperm functions.

## Introduction

All biophysiological events in cells involving enzymes, hormones, transmitters, and growth factors are dependent on pH. Any alteration in pH leads to either inhibition of function or deviation of cell function. Biological macromolecules have evolved to perform their function in specific cellular environment, and their dependency on pH for activity and stability reflects the significance of pH. Proton (and proton equivalent) has a crucial role in eukaryotic cellular function as all proteins depend on pH to maintain their structure and function. Along with these, every individual organelle in a cell is capable of performing a function on establishment of pH gradient [1].

Spermatozoa are specialized cells with sole purpose to fertilize the oocyte and deliver its genetic information to next generation. On ejaculation, millions of sperm cells are released into female reproductive tract from which only a few reach their target. To reach the target after being released from gonads, sperm has to encounter inconsistent extracellular environment with fluctuating concentration of ions, pH, pollutants, temperature, and other physiochemical variables which influence sperm behavior and metabolism [2]. Among these factors, pH could have a major initial effect on sperm activity and motility before as well as during the process of fertilization.

**Figure-1:**
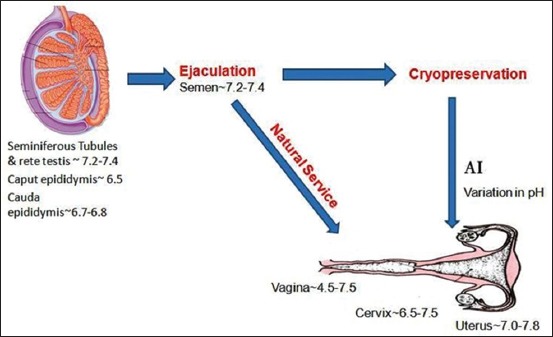
Different pH gradient in male and female genital tract implying a varying pH gradient for spermatozoa (Picture Courtesy: DKS, AKM and AK).

In mammalian seminiferous tubules and rete testis, sperm cells encounter pH of 7.2-7.4, which become 6.5 in caput epididymis and 6.7-6.8 in cauda epididymis [3,4]. During ejaculation, pH of semen becomes 7.2-7.4, and further after ejaculation into vagina, pH becomes 4.5-7.5, and in cervix, pH increases to 6.5-7.5 and ultimately in uterus and fallopian tube (7-7.8) (Figure-1) [3,4].

Sperm is the only cell whose activity is outside the male body, and in the inconsistent chemical milieu of sperm, seminal plasma may have profound effects on sperm quality and pH is one of the most critical factors which determine the semen quality. Spermatozoa are highly affected by pH. Functions such as motility, viability, capacitation, and acrosome reaction are pH dependent. During the journey in both male and female genital tract, sperm cell shows precise regulation of proton gradient and thereby regulation of intracellular pH. Studies have shown deviated/disrupted sperm functions at high/low pH indicating the existence of dynamic pH regulatory system in spermatozoa [5]. Some studies related to the effect of pH in birds, fish, shellfish, and mammals have been done, and nearly, all the results show the promising effect of pH on sperm motility, viability, and capacitation.

Significance of pH in regulating functional dynamics of spermatozoa begins from the testis (spermatogenesis) to their storage in epididymis and further its movement along the male reproductive tract [3]. During sperm cell journey, there is an interaction with glandular secretions and thereby modulation in sperm behavior. All these components of seminal plasma are regulated by a tightly controlled buffering system [5,6]. Spermatozoa are bathed in seminal plasma which is complex fluid secretions of accessory sex glands of male reproductive system and potentially contains all active component required for the optimal regulation of pH in and around sperm cells [4,6]. From these, it is evident that sperm functions are regulated by pH of genital tract of both male and female. This regulation is very precise and controlled by dynamic ion regulation along the sperm membrane [2].

## pH Regulatory Mechanisms in Different Animals

### Fish

Fishes basically are adapted to aquatic life and show external fertilization. Spermatozoa are passed into water where they swim to reach the site of fertilization, and in this prospective, importance of water salinity comes into picture. Salinity of water regulates its pH and depends on both soluble and insoluble particulates [7]. Alteration in water pH not only affects spermatozoa function but also makes the spermatozoa incompetent to bring fertilization [8]. Ion concentration, osmotic pressure, pH, temperature, and dilution of water affect sperm functional parameters [7].

pH has no direct effect on sperm function in fishes rather altered pH affects the endocrinological mechanisms associated with initiation of sperm motility. Altered pH in medium affects pituitary gonadotropin axis which remarkably alters the hormonal axis associated with the regulation of sperm functional dynamics [8]. Pituitary gland and gonadotropin stimulate the production of 17α-hydroxyprogesterone in testicular somatic cells which markedly stimulate the production of 17α, 2β-dihydroxy-4-pregnen-3-one in spermatozoa, and this results in increased pH in sperm duct as well as increase in intrasperm cyclic adenosine 3’,5’-monophosphate (cAMP) that initiates sperm motility. The hormone induced cAMP plays the cen tral role in regulating sperm motility [9].

### Bird

pH regulation in birds spermatozoa is a critical process and is highly significant as birds do not exhibit separate tract for urine and spermatozoa. Both excretory wastes and reproductive cells pass through the same tract. Uricotelism results in the excretion of uric acid in excretory tract but does not affect the reproductive cell indicating a potential role of pH-regulating system in avian spermatozoa. The exact role of pH along the sperm membrane is not only clear but also studies have indicated a potential role of pH on endocrine axis of birds and ultimately regulating sperm function [10-13]. Spermatozoa are stored for long period of time in sperm storage tubules in female birds. These structures also mediate sustained and prolonged release of spermatozoa to the site of fertilization. After their release, complex interaction with temperature, pH, ionic composition, and other factors occurs which regulate sperm motility. In domestic fowl, elevation in temperature from 30 to 40°C resulted in decrease motility, whereas alkalinization of external pH restored sperm motility at 40°C [10]. Similar results were shown in turkey and quail, in terms of velocity and percentage of motile spermatozoa which are increased in alkaline pH, and as compared to 30°C, 40°C requires more alkaline pH to initiate motility [11]. Body temperature (average 38.3°C) and slightly alkaline condition seem to stimulate ostrich sperm motility; this response is similar to the previous studies in avian species [12]. Mechanism responsible for the quiescent stage of motile sperm in sperm storage tubule is unclear, but in quail, low oxygen and high lactic acid concentration were identified, and by lowering the pH, motility was reduced [13].

### Mammals

Mammals exhibit most complex mechanisms to regulate pH in spermatozoa. Past studies have demonstrated that mammalian spermatozoa display complex mechanisms to regulate intracellular pH. The presence of Hv1channel (proton-gated channel) in mammalian spermatozoa has revealed the importance of H^+^ ion in regulating sperm functional parameters [14]. The precise regulation of H^+^ fluxing regulates intracellular sperm pH and also it associates with other ions to regulate pH. The presence of HCO_3_^−^ system further gives an idea regarding the complex mode of the regulation of pH [15]. It is also obvious that the movement of spermatozoa from testis to epididymis, in male and female reproductive tract encounters differential pH medium (as described earlier) but spermatozoa successfully reach the site of fertilization [14]. This gives rise to a dynamic concept of pH regulation in spermatozoa. The cytoplasmic pH of sperm could directly be affected by external pH of sperm with complex regulatory mechanism. Sperm intracellular pH exhibits a linear relationship with extracellular pH and studies have also shown that intracellular pH regulates ionic regulation along the sperm membrane [16].

Intracellular pH also regulates the opening of sperm-specific Ca^++^ channel (CatSper) in sperm flagella, and this is the major ion channel that regulates sperm intracellular Ca^++^. Processes such as sperm hyperactivation, capacitation, and acrosome reaction are also associated with intracellular Ca^++^ fluxing through CatSper channels [17,18]. Pharmacological and electrophysiological studies have revealed the precise role of pH (H^+^) in regulation as well as activation of CatSper channels and thereby regulation of intracellular Ca^++^. Progesterone-mediated Ca^++^ fluxing in spermatozoa is also governed by extrusion of H^+^ from spermatozoa due to the activation of Hv1 channels causing intracellular alkalization in spermatozoa [19].

Groundbreaking studies done by Babcock *et al*. [20] threw light on the role of internal pH in metabolism and motility of spermatozoa. They measured the cytosolic pH of bovine spermatozoa using fluorescein chromophore and showed that internal alkalization stimulates both motility and metabolism of epididymal bull spermatozoa. Intracellular pH is directly correlated with sperm motility, and in medium containing weak acids, sperm motility was suppressed as pH decreased. Reversible immobilization was established in this acidic medium, and after removal of weak acids, motility was restored up to 48 h in CO_2_ and up to 24 h in sperm dilution medium. This study revealed an inverse relationship between expression and conservation of sperm motility and depicts that intracellular acidification in spermatozoa suppresses motility and extend lifespan [21]. Studies in bovine spermatozoa have established the fact that intracellular pH substantially regulates sperm motility [20]. With the reduction in pH from 6.5 to 6, the sperm motility was decreased linearly indicating the potential role of pH in the regulation of sperm motility. Controlled sperm studies keeping viscosity and temperature of follicular fluid constant with a variable pH resulted in alterations in sperm motility and thereby indicated the potential role of pH in regulating sperm motility [22]. Kinematic study on the effect of pH on bull sperm function revealed the higher value of kinetic parameters, membrane integrity, and mitochondrial activity at pH 7 and 7.5, while pH lower than 6.5 and above 8 resulted in compromised motility with a decrease in most of the parameters. The higher pH (8.5) immobilized spermatozoa through a significant reduction in mitochondrial activity [23]. With a high pH, mitochondrial membrane potential decreases leading to the generation of the reduced amount of adenosine triphosphate (ATP). This results in reduced motility. Similarly, at an acidic pH (below 6.5), lower mitochondrial activity was observed leading to lower sperm motility. Optimal pH restores mitochondrial activity and thereby restores motility [23].

Analysis of internal pH of sperm in ram and boar in different ionic environment has shown a linear relationship between external pH and internal pH. High external pH increased intracellular pH and initiated motility in boar epididymal spermatozoa. However, in ram, motility of sperm was less dependent on external pH and effect was non-significant [16]. In buck, intracellular pH was increased with the alterations in extracellular pH using dialyzed epididymal plasma and bicarbonate, and this initiated forward motility in goat caput-epididymal sperm. When the pH of media was elevated from 7 to 8, about 55% cauda epididymal sperm became motile [24]. Motility of cauda epididymal sperm was studied in different pH in the presence and absence of motility inhibitory factor (MIF II). Lower pH (4.0-4.5) suppressed the motility and optimum pH for MIF II activity was observed between pH 6.5 and 7.5; in alkaline pH above 7.5, MIF II activity was vanished [25]. Sperm forward motility promoting potency of motility initiating protein (MIP) was maximal at pH 8 while neutral pH caused little activity of MIP [26]. Both the inhibitory factors and initiating protein show pH dependency for their activity.

Like other mammals, human spermatozoa also show dependency on pH for its functional activity [14]. Before fertilization, spermatozoa undergo capacitation and develop the ability to respond to the inducers of acrosome reaction and this development of acrosomal responsiveness is very much affected by pH [5,27]. Studies have demonstrated that the internal pH had a positive relationship with external pH. The d ecrease in external pH lowered the cytoplasmic pH and subsequently reduced the acrosomal responsiveness [5,16]. Although unesterified cholesterol of sperm must be lost in the process of acrosome reaction, the loss of cholesterol did not get affected by variation in pH [28]. When healthy human spermatozoa were cultured in sperm nutrition solution with varying pH, sperm viability, motility, and hypoosmotic swelling rate were maximum at pH 7.2 and decreased in more acidic and more basic medium [27]. Sperm Na^+^/K^+^-ATPase activity was lower at pH 5.2, 6.2, 8.2, 9.2, and 10.2 as compared to pH 7.2. Alkalization of medium increased intracellular Ca^2+^ required for motility and capacitation [27]. Progressive motility and viability of sperm were decreased in acidic medium (pH 5.2 and 6.2), sperm penetration was higher at pH 7.2 along with Na^+^/K^+^-ATPase activity, and Ca^2+^ influx decreased significantly in acidic environments [5].

**Figure-2:**
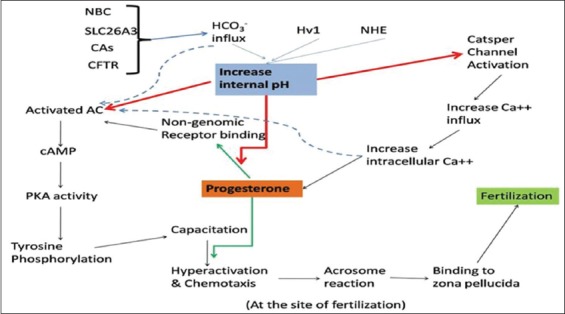
Schematic insight to regulation of pH in spermatozoa as well as dynamic signaling pathways in spermatozoa in terms of pH-dependent mechanism. Appropriate intracellular pH is required for sperm functions to achieve successful fertilization to achieve successful fertilization. Regulation of Η+ influx is mediated by HCO3−, influx. Voltage-gated proton channel, Na+/H+ exchanger Na+/HCO3−co-transport, and CI−/HCO3− exchanger (SLC26A3) are responsible for HCO3− influx, so Na+ and CI− are indirectly involved in pH regulation. Progesterone hormone binds to non-genomic receptor and is dependent on pH. With influx of HCO3− and Ca++, enzyme-soluble adenylyl cyclase is activated which leads to increased generation of cyclic adenosine 3’,5’-monophosphate and subsequent increased protein kinase A (PKA) activity. Increased PKA activity leads to phosphorylation of tyrosine-containing protein on spermatozoa which are the key players behind the induction of capacitation/hyperactivation. Eventually, these events mediate interaction of spermatozoa with oocyte resulting in fertilization. The action of progesterone is pH dependent and occurs in a dose-dependent manner (Picture Courtesy: AKM, AK and DKS).

Series of *in vitro* studies have documented as well as established the fact that pH is a significant player in regulating vital physiological functions of spermatozoa [5,12,13,23]. Studies also have indicated the existence of specific, accurate, and tightly regulated system to functionally regulate pH in spermatozoa [4,14,15]. pH regulation is also precise, and there may be the existence of other systems like ion channel regulatory systems to regulate pH in spermatozoa [2,14,17,19]. Figure-2 depicts the role of pH and its associated mechanism regulating sperm physiological functions.

### pH regulation

Intracellular alkalinization is essential for triggering several physiological responses which are crucial for fertilization, and this proposes the existence of mechanisms to control internal pH [16]. The mechanism involved in proton transfer from cytoplasm across the sperm plasma membrane to extracellular environment is a species-specific event and along with it varies from cell to cell [2,14]. Due to the involvement of cells and species-specific mechanisms, the mechanisms of internal alkalization are still a topic of interest and investigation.

**Figure-3:**
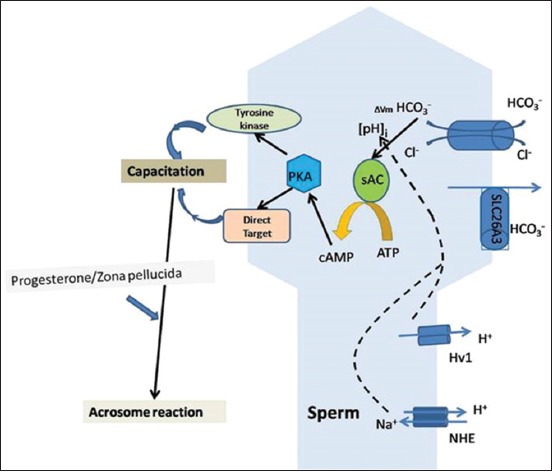
Regulation of sperm intracellular pH through three different mechanisms (Picture Courtesy: AKM, AK and DKS).

pH regulation in spermatozoa is regulated by three mechanisms, namely, HCO_3_^−^ influx, voltage-gated proton channel (Hv1), and Na^+^/H^+^ exchanger (NHE). These three mechanisms have been extensively evaluated using molecular, pharmacological, and electrophysiological tools, and the studies have hypothesized the existence of these systems independently in spermatozoa and are regulated by different mechanisms (Figure-3).

### HCO_3_^-^ influx system

HCO_3_^−^ influx system involves the inward movement of HCO_3_^−^ which is essential for sperm capacitation. It produces cAMP by activating soluble adenylate cyclase (sAC) and promotes alkalinization and membrane hyperpolarization [29]. When Na^+^ is removed from the medium, then this alkalinization and hyperpolarization did not occur, indicating the presence of Na^+^/HCO_3_^−^ cotransport mechanism in spermatozoa [15]. HCO_3_^−^ entrance in sperm cytoplasm requires Cl^−^ and sperm capacitation depends on both Cl^−^ and HCO_3_^−^ concentration [29].

Carbonic anhydrase (CA) catalyzes the reaction of hydration of CO_2_ and produces HCO_3_^−^ and regulates the homeostasis of HCO_3_^−^ in sperm. With the onset of puberty in mice, CA II and CA IV are expressed along the epididymal epithelium; CA II is located in principle piece of sperm and CA IV is present in the plasma membrane of entire sperm tail [30]. Experimentation has shown that CA II and CA IV knockout mice have imbalance HCO_3_^−^ homeostasis [30].

Cystic fibrosis transmembrane conductance regulator (CFTR) controls the HCO_3_^−^ entrance-dependent events in human sperm capacitation. Inhibition of CFTR affected HCO_3_^−^ entrance adversely and subsequently reduced the activation of cAMP/protein kinase A (PKA) pathway, demonstrating that CFTR and PKA activities are crucial for regulation of intracellular pH [31].

### NHE

This mechanism involves proton transfer through NHE. Sperm-specific NHE is located in the flagellum and NHE-null mice were found sterile [32]. After leaving epididymis, spermatozoa encounter high Na^+^ concentration in seminal fluid which favors the exchange of anions across the membrane. When cell-permeable cAMP analogs were added in NHE-null spermatozoa, then it restored protein tyrosine phosphorylation activity and sperm motility in defective sperm suggesting the role of NHE in regulation of internal pH [32]. Male mice lacking NHEs (*NHA1* and *NHA2*) gene are sterile with diminished sperm motility. When treatment of NHA1 antisera was given to mice, reduced sperm internal pH was observed and it caused immobility probably through attenuating sAC-mediated cAMP synthesis [33].

### Hv1

Hv1 channel is an H^+^ transporter across the membrane and composed of a voltage sensor domain homologous to the voltage sensor of voltage-gated cation channel [34]. In contrast to a conventional ion channel, Hv1 lacks a classical pore region [35]. Although Hv1 molecule dimerizes, each subunit can function independently as a Hv1 [36]. Hv1 was identified in human spermatozoa and abundantly localized in the principal piece of sperm flagellum, which is the ideal position to activate pH-dependent protein of axoneme and thus to control sperm motility [37]. Hv1 is characterized by strong voltage dependence, activation by low intracellular pH, unidirectional proton extrusion, inhibition by a low concentration of zinc, and potentiation by fatty acids [14]. Sperm Hv1 conducts proton much more rapidly and efficiently and conducts them unidirectionally to the extracellular space [38]. Hv1 channel is involved in the regulation of sperm internal pH, and through this, almost every aspect of sperm function is get influenced in the female reproductive tract, including initiation of hypermotility, capacitation, hyperactivation, and acrosome reaction [14,37]. Sperm Hv1 can be activated by the removal of extracellular zinc, which is present in seminal plasma [39]. In female reproductive tract, any bound zinc is released through dilution or absorption by the uterine epithelium and chelation by albumin and other molecules cause the activation of Hv1 channel [40]. The low micromolar concentration of the endogenous cannabinoid anandamide strongly potentiates the sperm Hv1 [19].

### pH and infertility

Mammalian fertilization is intricately regulated event comprising several known and unknown factors such as genetic, physiological, environmental, and managerial factors [2]. Determination of the exact causes of infertility is difficult due to its multi-factorial complex interaction with other factors. As mentioned in previous section, pH is a key regulator in sperm physiology and function, suggesting that improper pH homeostasis could be one of the reasons for unexplained infertility [5]. Usually, vaginal pH is acidic and in human ranges from 4.0 to 4.9, and in other non-human mammals, vaginal pH is 6.8 [3,41]. Fluctuation in vaginal pH is observed which is mainly due to vaginal microbiome and hormonal variation in reproductive cycle. Lactic acid producing *Lactobacilli* and peak estrogen level are lower the vaginal pH [41]. The alkaline pH of semen defends sperm in acidic environment so inadequate semen volume or decreased alkalinity of seminal vesicular fluid may affect buffering capacity and result in compromised fertility [6]. Variation in vaginal pH makes reproductive tract more susceptible for infection, and inflammatory responses have been observed in infertile females with abnormal vaginal flora [42]. Colonization of sperm-agglutinating *Staphylococcus aureus* has been reported to cause immobility in spermatozoa [43].

Kidney disorders due to various diseases, poisoning of drugs, severe dehydration, and subacute ruminal acidosis in cattle could cause metabolic acidosis which brings down blood pH (7.4) [44,45]. Chronic metabolic acidosis is associated with increased cortisol secretion [46], and this stress-induced rise of cortisol exerts inhibitory effect on the secretion of LH and testosterone which interfere with normal sperm production in testis. Ultimately, this chronic acidosis could be the reason for male infertility [45].

Development of artificial insemination (AI) technique is a boon in reproductive science and has been used intensively to boost the reproduction. Livestock farms, especially bovine, have used AI as their favorite tool for increasing production. After the collection of semen from superior sire, semen undergoes cryopreservation, and before preservation, semen is diluted with dilutors. Diluent pH is very much important for the maintenance of sperm respiration and motility. To counter pH changes, buffering agents are added to dilutors, and therefore, improper pH maintenance during freezing could affect efficiency of AI adversely [47].

Bacterial contamination is an inevident process during semen collection and processing of ultralow freezing. Microbial contamination results in a reduction in the pH of the diluents as well as this pH affect the overall quality of sperm cells. Studies have a shown significant reduction in sperm motility and viability in acidic pH during microbial contamination [48]. Due to low pH, the post-thaw quality of spermatozoa also gets reduced and resulted in poor or compromised conception rates [49,50]. pH alteration is also associated with the alteration in medium osmolarity, and that is why functional alterations have been observed during freezing and thawing [50]. Therefore, in the context of semen freezing and AI, pH of the medium, as well as pH during storage, plays a significant role in regulating sperm functional dynamics.

## Conclusion

Precise regulation of sperm functional parameters in context to pH is still under investigation. Rapid changes in the external environment during sperm journey to oocyte are dynamically regulated by ion channels. pH plays a significant role in regulating sperm motility and fertility competence. That is why spermatozoa have developed dynamic pH regulatory systems by which there is a regulation of intracellular pH. Intracellular pH is affected by extracellular pH and thereby opens many windows of investigation regarding the role of pH in functional significance of spermatozoa.

Increasing evidence of human male infertility in diabetic acidosis cases has raised many questions regarding the role of pH in spermatozoa functions. The multimeric approach is required to solve the mysteries behind pH and sperm functions. Understanding pH and its role in regulating spermatozoa function will help to develop strategies to understand and treat the basic causes behind infertility associated with pH.

Cryopreservation-associated reduction in osmolarity and pH are critical regulators of sperm attributes after thawing. Altered pH has a negative effect on sperm functional competence. The use of buffers in dilutors solves these issues somehow, but still, it needs more improvement. Understanding the role of pH in the regulation of spermatozoa functions will help in the development of suitable agents which will restore pH during freezing and thawing.

## Author’s Contributions

AKM and AK designed and framed the manuscript as a part of their research under the supervision of DKS. RN and SY carried out the proofreading and finalized the manuscript and guided entirely during the preparation of this manuscript. DKS designed the concept and finalized the manuscript for publication. AKM, AK and DKS designed the figures for this review. All authors read and approved the final manuscript.
